# ­­Statins in the perioperative period

**DOI:** 10.12688/f1000research.17572.1

**Published:** 2019-05-20

**Authors:** Reza Mohebi, Robert Rosenson

**Affiliations:** 1 Department of Medicine (Cardiology), Icahn school of Medicine at Mount Sinai, New York, 10029, USA

**Keywords:** Statin, perioperative risk, mortality, risk reduction

## Abstract

In this review, we discuss clinical evidence-based data regarding the potential benefit of statin therapy in the perioperative period of non-cardiac surgery. Results from meta-analyses of prospective observational studies have provided conflicting evidence. Moreover, comparison among studies is complicated by varying data sources, outcome definitions, types of surgery, and preoperative versus perioperative statin therapy. However, results of two recent large prospective cohort studies showed that statin use on the day of or the day after non-cardiac surgery (or both) is associated with lower 30-day all-cause mortality and reduction in a variety of postoperative complications, predominantly cardiac, compared with non-use during this period. There is a paucity of data from randomized controlled trials assessing the benefit of statin therapy in non-cardiac surgery. No randomized controlled trials have shown that initiating a statin in statin-naïve patients may reduce the risk of cardiovascular complications in non-cardiac surgeries. One randomized clinical trial demonstrated that the use of a preoperative statin in patients with stable coronary heart disease treated with long-term statin therapy had a significant reduction in the incidence of myocardial necrosis and major adverse cardiovascular events after non-cardiac surgery. In conclusion, it is important that all health-care professionals involved in the care of the surgical patient emphasize the need to resume statin therapy, particularly in patients with established atherosclerotic cardiovascular disease. However, initiating a statin in statin-naïve patients undergoing non-cardiac surgery needs more evidence-based data.

## Introduction

Many patients undergoing surgery take medicines used to prevent atherothrombotic cardiovascular events. The health-care impact of temporary discontinuation of cardiovascular preventive therapies on perioperative and postoperative complications is an important concern for physicians involved in preoperative cardiovascular risk assessment ^1^. Cardiovascular complications, including myocardial infarction, acute congestive heart failure, atrial fibrillation, fatal ventricular arrhythmia, and cardiac death, were found in up to 5% of patients undergoing in-hospital non-cardiac surgery ^2, 3^. Of these complications, perioperative myocardial infarction is the most common, occurring in 16% of patients with cardiac complication ^4^. Large prospective cohort studies have shown that the incidence of primary myocardial injury following non-cardiac surgery (MINS) ranges between 8 and 19% and that myocardial infarction accounts for about 40% of myocardial injury ^5– 7^. In this respect, cardiovascular risk assessment and optimization of medical therapy play important roles for risk reduction of adverse complications of non-cardiac surgeries ^8^.

Hydroxymethylglutaryl coenzyme A reductase inhibitors (statins) have been used since the mid-1980s. The use of statins in adults in the US has increased substantially in the last decade ^9^. Tens of millions of individuals have received statins as therapy for the primary and secondary prevention of coronary atherosclerotic events by lowering of lipid levels ^10^. Furthermore, use of statins offers beneficial effects beyond those afforded by reducing low-density lipoprotein cholesterol (LDL-C) levels. It is postulated that the pleiotropic effects of statins, which can change endothelial function, decrease inflammation ^11^, and alter membrane receptors/ion channels ^12^, may also have beneficial effects in individuals undergoing non-cardiac surgeries ^13, 14^. In this review, we discuss evidence-based data concerning the continued use of statins in the perioperative period.

## Discussion

There is a paucity of data from randomized clinical trials assessing the benefit of statin therapy in non-cardiac surgery ( Table 1). In the Lowering the Risk of Operative Complications Using Atorvastatin Loading Dose (LOAD) randomized trial ^15^, reported by Berwanger *et al*., 648 statin-naïve patients who were at risk for a major vascular complication and scheduled for non-cardiac surgery were randomly assigned to a loading dose of atorvastatin or placebo (80 mg anytime within 18 hours before surgery) followed by a maintenance dose of 40 mg (or placebo), started at least 12 hours after surgery, and then 40 mg (or placebo) daily for 7 days. A composite of all-cause mortality, non-fatal MINS, and stroke at 30 days was the primary outcome. In contrast to prior observational cohort studies, the LOAD trial did not show any risk reduction in major cardiovascular complications after a short-term perioperative course of statins in statin-naïve patients undergoing non-cardiac surgery. Also, the results of the Dutch Echocardiographic Cardiac Risk Evaluation Applying Stress Echocardiography IV (DECREASE-IV) randomized controlled trial ^16^ demonstrated that patients who had an intermediate cardiovascular disease risk—which was defined by an estimated risk of perioperative cardiac death and myocardial infarction of 1 to 6% by using clinical data and type of surgery—and who were randomly assigned to fluvastatin experienced a lower incidence of the end point than those randomly assigned to fluvastatin-control therapy (3.2% versus 4.9% events; hazard ratio 0.65, 95% confidence interval [CI] 0.35–1.10), but statistical significance was not reached ( *P* = 0.17). Moreover, in regard to the effect of statins in non-cardiac vascular surgeries, a Cochrane review pooled the results from three vascular surgery trials including 178 participants and found no difference in decreasing the risk of mortality and myocardial infarction at 30 days with statins ^21^.

**Table 1.** Descriptive baseline characteristics and findings of studies.

Study	Year	Number of patients	Patient population	Type of surgery	Follow-up duration	Statin effect on adverse outcome
Clinical trials						
LOAD trial	2016	648	High-risk statin-naïve patients	Non-cardiac surgery	30 days	No risk reduction
Xia *et al*. ^17^	2015	550	Stable coronary artery disease on long-term statin	Non-cardiac	6 months	Risk reduction
DECREASE-IV	2009	1066	Patients at intermediate cardiac risk	Non-cardiac	34 days	No reduction
Meta-analysis						
Hindler *et al*. ^18^	2006	223,010 (15 studies)	General patients	General surgery including cardiac	-	Risk reduction
Antoniou *et al*. ^19^	2015	22,681 (24 studies)	General patients	Vascular surgery	-	Risk reduction
Observational cohort						
London *et al*. ^20^	2017	180,478	General patients	Non-cardiac surgery	30 days	Risk reduction
VISION cohort	2016	15,487	General patients	Non-cardiac surgery	30 days	Risk reduction

DECREASE-IV, Dutch Echocardiographic Cardiac Risk Evaluation Applying Stress Echocardiography IV; LOAD, Lowering the Risk of Operative Complications Using Atorvastatin Loading Dose; VISION, Vascular Events in Noncardiac Surgery Patients Cohort Evaluation.

In contrast to these two studies, a single-center, double-blind, placebo-controlled trial in China showed that, in patients with stable coronary heart disease on long-term statin therapy, preoperative rosuvastatin therapy decreases the incidence of myocardial necrosis and major adverse cardiovascular events after non-cardiac surgery ^17^. Moreover, a prospective study showed that discontinuation of statins in patients with a previous myocardial infarction is associated with higher rates of recurrent myocardial infarctions and more hospitalizations for cardiovascular events than patients highly adherent to statin therapy ^22^.

Results from a meta-analysis of prospective observational studies provided conflicting evidence ^23^. A comparison of studies is complicated by varying outcome definitions, types of surgery, and preoperative versus perioperative statin use. A prospective cohort study analyzed 15,478 patients from the Vascular Events in Noncardiac Surgery Patients Cohort Evaluation (VISION) trial ^24^ who were at least 45 years old and had non-cardiac surgery; in the matched population of 2845 patients (18.4%) who received a statin and 4492 (29.0%) controls, preoperative statin use was associated with a lower risk of the primary outcome, a composite of all-cause mortality, MINS, or stroke at 30 days (relative risk 0.83, 95% CI 0.73–0.95). Statins were also associated with a significantly lower risk of the individual components of all-cause mortality, cardiovascular mortality, and MINS, but there were no statistically significant differences in the risk of myocardial infarction or stroke. Recently, London *et al*. ^20^, in a retrospective observational cohort, analyzed a large database of 180,000 veterans who were undergoing elective or emergent non-cardiac surgery and who were admitted within 7 days of surgery and underwent 30-day postoperative follow-up. The analysis results indicate that statin use on the day of or the day after non-cardiac surgery (or both) was associated with lower 30-day all-cause mortality and reduction in many postoperative complications, including cardiac complications, compared without receiving statin during this period. Moreover, sub-analyses demonstrated that patients younger than 75 years, those receiving intensive statin therapy, patients with ischemic heart disease or diabetes, and those undergoing high-risk surgery may have a larger risk reduction with perioperative statin treatment. Discontinuation of perioperative statin treatment may increase the risk of adverse outcomes.

## Conclusions

A protective effect of statin therapy in patients undergoing non-cardiac surgeries has been reported in many prospective observational studies. However, data from randomized controlled trials showing the effect of a perioperative course of statins for non-cardiac surgery are inconsistent. Of the three trials published in the field, two (LOAD and DECREASE-IV) failed to show a protective effect of statin therapy in the preoperative period. The third trial showed a protective effect of statin therapy only in those who had stable coronary heart disease and received statins over a long period of time. In conclusion, as a quality measure, it is important that all health-care professionals involved in the care of the surgical patient emphasize the need to resume statin therapy, particularly in patients with established atherosclerotic cardiovascular disease. However, initiating statin therapy in statin-naïve patients undergoing non-cardiac surgery needs further randomized controlled trials.
